# Genome-Wide Characterization and Expression Analysis of Transcription Factor Families in Desert Moss *Syntrichia caninervis* under Abiotic Stresses

**DOI:** 10.3390/ijms24076137

**Published:** 2023-03-24

**Authors:** Haron Salih, Wenwan Bai, Mingqi Zhao, Yuqing Liang, Ruirui Yang, Daoyuan Zhang, Xiaoshuang Li

**Affiliations:** 1 State Key Laboratory of Desert and Oasis Ecology, Key Laboratory of Ecological Safety and Sustainable Development in Arid Lands, Xinjiang Institute of Ecology and Geography, Chinese Academy of Sciences, Beijing 100045, China; 2 Xinjiang Key Laboratory of Conservation and Utilization of Plant Gene Resources, Xinjiang Institute of Ecology and Geography, Chinese Academy of Sciences, Urumqi 830000, China; 3 University of Chinese Academy of Sciences, Beijing 100049, China

**Keywords:** transcriptional regulation, desert moss, gene expression levels, cold stress, dehydration-rehydration stress, protein interaction

## Abstract

Transcription factor (TF) families play important roles in plant stress responses. *S. caninervis* is a new model moss for plant desiccation tolerance studies. Here, we report a high-confidence identification and characterization of 591 TFs representing 52 families that covered all chromosomes in *S. caninervis*. GO term and KEGG pathway analysis showed that TFs were involved in the regulation of transcription, DNA-templated, gene expression, binding activities, plant hormone signal transduction, and circadian rhythm. A number of TF promoter regions have a mixture of various hormones-related *cis*-regulatory elements. AP2/ERF, bHLH, MYB, and C2H2-zinc finger TFs were the overrepresented TF families in *S. caninervis*, and the detailed classification of each family is performed based on structural features. Transcriptome analysis revealed the transcript abundances of some Sc*AP2/ERF*, *bHLH*, *MYB*, and *C2H2* genes were accumulated in the treated *S. caninervis* under cold, dehydration, and rehydration stresses. The RT-qPCR results strongly agreed with RNA-seq analysis, indicating these TFs might play a key role in *S. caninervis* response to abiotic stress. Our comparative TF characterization and classification provide the foundations for functional investigations of the dominant TF genes involved in *S. caninervis* stress response, as well as excellent stress tolerance gene resources for plant stress resistance breeding.

## 1. Introduction

Abiotic stress negatively impacts plant growth and development, as well as poses a significant threat to plant production and food supply [1]. Plants respond to periods of abiotic stress by motivating specific physiological, biochemical, and molecular elements to mitigate the undesirable impact of the abiotic stress combination on plant growth and developmental stages [2,3]. Transcription factors (TFs) are key regulators in gene expression, transcriptional regulation, and signal transduction pathways by recognizing specific *cis*-regulatory DNA sequences in the promoters of downstream genes [4,5]. TFs contain four domains: a DNA-binding, transcription activation or repression, a nuclear localization indicator, and an oligomerization region [6]. These four domains systematically act together to regulate transcriptional and posttranscriptional processes by activating or repressing gene expression patterns in response to external and internal stimuli during plant growth and developmental phase [7]. TFs and their binding sequences significantly interact with each other and help plant cells respond to diverse environmental conditions [8,9].

In plants, a large number of recognized TFs ranged from 6% to 10% [10]. In the PlantTFDB database, a total of 320370 TFs were identified from 165 species and classified into 58 families, which cover the main lineages of green plants and provide genomic TF repertoires across green plants [11]. In the model plant *Arabidopsis*, which has 1770 TFs in its genome, these TFs can be classified into different families based on their structure and binding domains [12]. In recent years, many studies have shown that numerous plant TFs, including the members of APETALA2/ethylene-responsive element binding factors (AP2/ERF), myeloblastoma (MYB), basic helix-loop-helix (bHLH), NAM, ATAF1/2, CUC2 (NAC), basic leucine zipper (bZIP), and C2H2-zinc finger genes, are significantly involved in plant stress responses [13,14]. Genome-wide comparative analysis of all the TFs in plant species is a good strategy for identifying valuable stress-tolerant genes [12]. As high-throughput sequencing technology advances, the availability of many plant genome sequences provides an important opportunity for investigating the genome-wide characterization of TF families. Many important TF family members were identified in different plant species based on the whole genome dataset from various lineages. A huge number of TF genes were detected in Arabidopsis (1770), rice (1801), and *V. vinifera* (1659) [15]. In bryophytes, a total of 1078, 423, and 333 TF members were initially identified in the *P. patens*, *M. polymorpha*, and *A. angustus* genomes, respectively [15]. In algae, *V. carteri* genome has 125 TF genes and *K. flaccidum* genome has 232 TF genes [16].

*Syntrichia caninervis* is a dominant species of biological soil crust in the Gurbantunggut Desert of Northwestern China [17]. *S. caninervis* is a desiccation-tolerant moss, which can survive equilibration with extremely dry air (i.e., 0–30% RH or less than −162 MPa) [18]. It has emerged as an ideal model for understanding the molecular mechanism of plant desiccation tolerance as well as a potential source of stress-related gene identification for crop improvement [19,20,21,22]. In the previous study, based on the dehydration-rehydration transcriptome of *S. caninervis*, we identified a total of 778 TFs [23] and cloned several TFs in *S. caninervis*, such as ScDREB5, ScDREB8, ScDREB10, ScABI4, and ScABI3, which play important roles in DT responses. The most gratifying discovery is that, unlike the TF genes isolated from Arabidopsis and other angiosperms, the TF genes cloned from *S. caninervis* also exhibited significantly improved drought/salt/cold/heat stress tolerances in Arabidopsis without growth inhibition [19,24,25,26,27]. All of these studies have demonstrated that *S. caninervis* stress-tolerant TFs are excellent candidate genes for plant stress-tolerance molecular breeding [28].

Recently, the genome data of *S. caninervis* has become available [29], but there has yet to be systematic research on the high-throughput mining of stress tolerant-related TFs in the *S. caninervis* genome. In addition, as a representative species that has evolved from aquatic to terrestrial, relatively little is known about the functional role and evolution information of TFs in moss responses to abiotic stress. A comprehensive analysis of *S. caninervis* TFs and their evolutionary functions might help to elucidate the regulatory mechanism underlying stress responses. Here, we carried out a comprehensive genomic analysis of TFs in *S. caninervis*, which identified that 591 TF members encoded genes. All TFs were consequently subjected to systematic analysis, including conserved domains, chromosomal positions, gene ontology (GO), KEGG pathway enrichment, regulatory information, phylogeny tree analysis, network interactions, comparison studies of TF numbers across various species, and gene expression patterns under cold exposure and dehydration-rehydration (D-R) stress. The findings of this work will provide fundamental information about the TFs in *S. caninervis*, as well as contribute to future investigations of the functions and regulatory mechanisms of dominant TF genes involved in *S. caninervis* stress responses. Additionally, global identification and comparative analysis of TF genes from related lineages extend our knowledge of TF evolution and function.

## 2. Results

### 2.1. Identification of S. caninervis TFs

**Figure 1:**
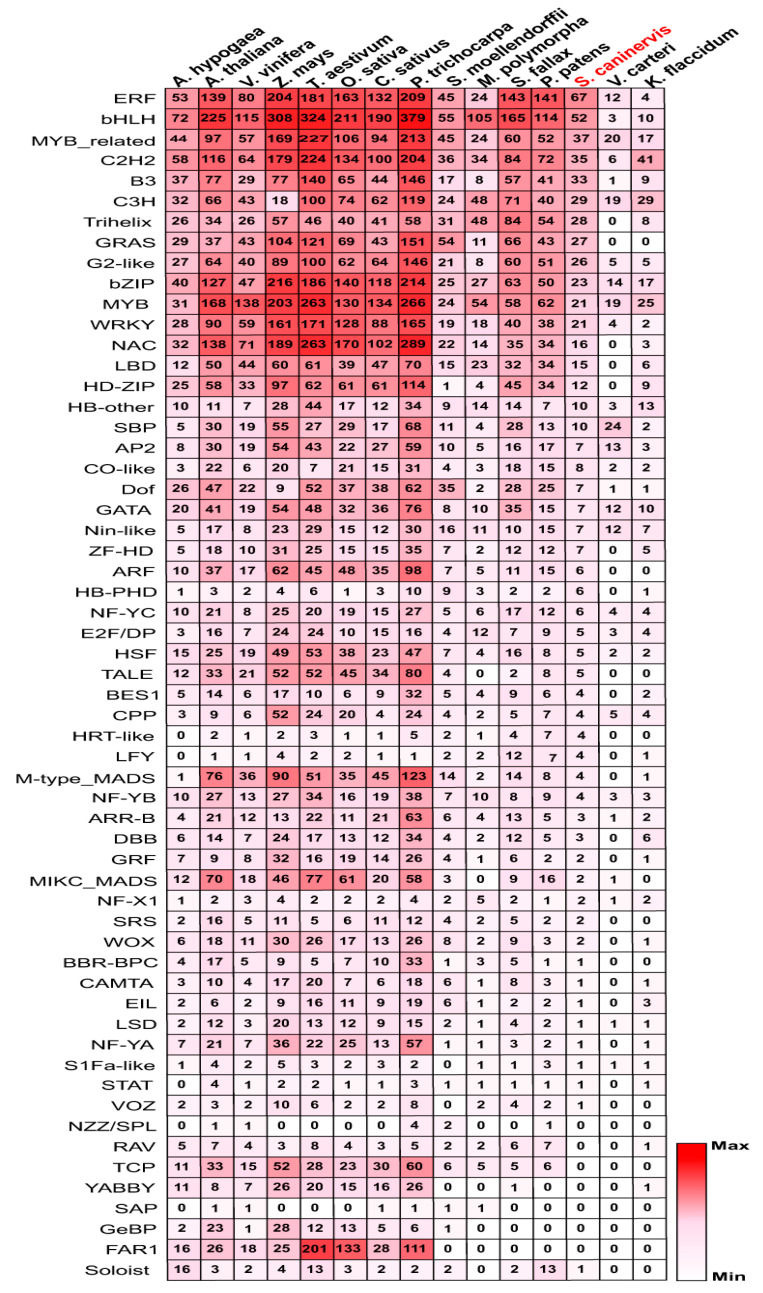
A list of different members of 591 TF families was observed in 14 represented plant species. The red color indicated the maximum number of TFs in different plant species. *S. caninervis* was labeled in red color.

Approximately 820 putative TF sequences were associated with the *S. caninervis* genome by blast searches. SMART database and InterPro online tools were employed to determine the existence of the TF domain features in *S. caninervis* protein sequences (Figure S1 and Table S1). The results of identification and characterization were improved by adjusting the assignment rules and prediction cutoffs of TF families in *S. caninervis*. Finally, a total of 591 (591/16,545 (3.6%)) non-redundant TFs belonging to 52 TF families were identified in *S. caninervis* (Figure 1 and Table S1). In addition, over-represented TFs include the AP2/ERF with 75, MYB (MYB/MYB-related) with 58, bHLH with 52, C2H2 with 35, B3 with 33, C3H with 29, Trihelix with 28 genes. Whereas 10 identified TF families with a single gene copy in the *S. caninervis* genome database, such as BBR-BPC VOZ and others (Figure 1 and Figure S1). Furthermore, the *S. caninervis* genome lacks TCP, FAR1, GeBP, SAP, RAV, NZZ/SPL, and YABBY families (Figure 1), suggesting that these TF family members may evolve after whole genome duplication. In this study, different members of TFs were identified in 14 plant species that belong to 58 TF families (Figure 1). AP2/ERF is one of the biggest families, with 75, 178, and 167 AP2/ERF genes potentially found in *S. caninervis*, *P. patens*, and *S. fallax*, while 31, 25, and eight genes were identified in *M. polymorpha*, *V. carteri*, and *K. flaccidum*, respectively (Figure 1). In *S. caninervis*, AP2/ERF TF superfamily includes three subgroups, AP2, ERF (ERF/DREB), and soloist subfamilies with 7, 67, and one gene, respectively. The proportions of the ERF subfamily (67/75 = 89.33%) were relatively expanded, and the percentages of the AP2 subfamily (7/75 = 9.33%) decreased in *S. caninervis.* In comparison with the proportions of these genes in *A. thaliana* (77.65% ERF: 16.75% AP2), *P. trichocarpa* (75.46% ERF: 21.93% AP2), *O. sativa* (80.23% ERF: 15.69% AP2), *Z. mays* (73.27% ERF: 21.86% AP2), *V. carteri* (48 % ERF: 52% AP2), *K. flaccidum* (50.0% ERF: 37.5% AP2), and *C. zofingiensis* (50% ERF: 50% AP2) (Table S2). According to the number of MYB repeats in protein sequences, 58 *S. caninervis* MYB genes were identified and classified into three distinct subgroups, 37 MYB-related (MYB-R1), 19 MYB-R2, and two MYB-R3 (Table S3). Similar results were found in *K. flaccidum*, *V. carteri*, potato, *B. distachyon*, and pineapple (Table S3). However, in other plants such as *P. patens*, *D. catenatum*, and *Arabidopsis*, MYB proteins are divided into four subgroups, MYB-related, MYB-R2, MYB-R3, and MYB-R4. MYB-related genes predominate in *S. caninervis*, but in higher plants, MYB-R2 is more common [30]. In fact, the numbers of the identified MYB TFs in different plant species gradually increased from algae and mosses to flowering plants (Table S3). The basic helix–loop–helix (bHLH) TF is characterized by a basic helix–loop–helix domain with two parts, a basic DNA binding domain at the N-terminal and a protein interaction domain at the C-terminal. Comparative results indicated that bHLH TFs in various plant species have increased gradually from algae to mosses and higher plants. For example, three species of algae genomes (*K. flaccidum*, *V. carteri*, and *C. zofingiensis*) have 18 members, *S. caninervis* has 52 members, *P. patens* has 114 members, and *O. sativa*, *A. thaliana*, *Z. mays*, and *P. trichocarpa* genomes have 211, 225, 308, 379 gene members, respectively (Figure 1). The C2H2-zinc finger protein family is the fourth most abundant TF family in *S. caninervis* with 35 genes. Different numbers of C2H2-zinc finger genes were found in 14 plant species (Figure 1).

### 2.2. Chromosomal Mapping of TF Genes in S. caninervis

**Figure 2:**
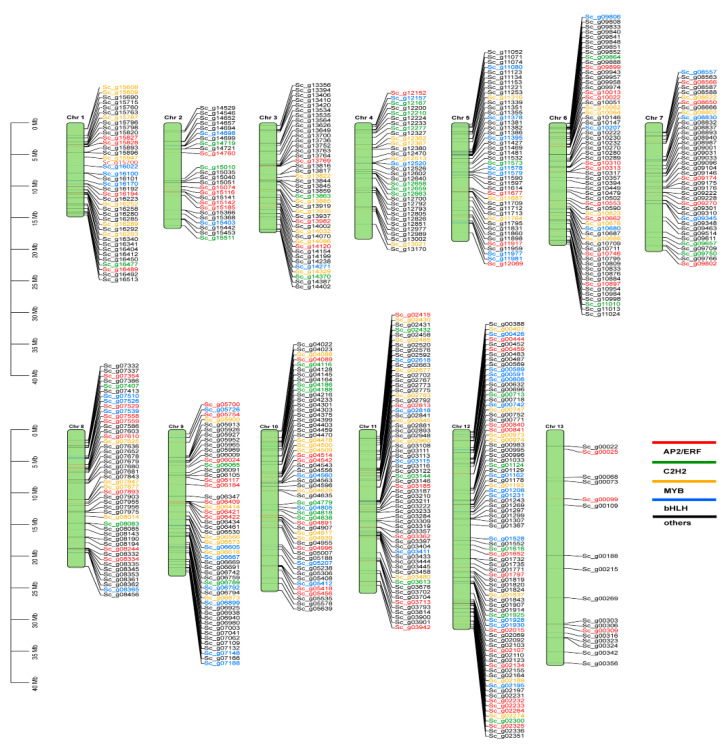
Chromosomal mapping of TF members on the *S. caninervis* genome based on their physical positions. The four most abundant TF families including, AP2/ERF, MYB, bHLH, C2H2, and others, were represented by different colors, such as red, green, orange, blue, and black, respectively.

Chromosomal position analysis of the *S. caninervis* genome found that 591 candidate TFs were assigned to all chromosomes and mapped according to their chromosomal locations. The distribution and density of TF genes in the chromosomal regions were not identical in the *S. caninervis* genome (Figure 2). Chromosomes 6, 11, and 12 harbored the highest numbers of TFs, whereas the lowest density of TFs (16 TFs) was mapped to chromosome 13 (largest chromosome) as shown in Figure 2. The detailed distributions of four over-represented TF families, including AP2/ERF, MYB, bHLH, and C2H2 were investigated. Seventy-five AP2/ERF genes covered all *S. caninervis* chromosomes. The highest numbers of AP2/ERF TFs were found on chromosome 12, with 13 TFs, followed by chromosome 6 with nine TFs, and chromosomes 8 and 9 with eight TFs for each. However, the lowest density of AP2/ERF members was detected on chromosomes 3, 13, and 4 with three, two, and one TF, respectively (Figure 2 and Table S1). A total of 52 bHLH TFs were straight-mapped to 12 *S. caninervis* chromosomes with unequal distributions (Figure 2). Chromosome 12 had 12 bHLH genes, followed by chromosomes 5 and 9 with seven bHLH genes for each, while the lowest bHLH numbers were found in chromosomes 2 and 3 with three genes for each. Fifty-eight MYB TF genes were anchored onto 11 chromosomes (Figure 2 and Table S1). Chromosome 12 had eight genes, followed by chromosomes 1, 10, and 11 each with seven genes (Figure 2). However, chromosomes 5 and 7 had the lowest numbers of MYB genes, with five genes. Thirty-five C2H2-zinc finger genes were mapped to 12 chromosomes (Figure 2 and Table S1). Chromosomes 10 and 4 each had six genes, but chromosomes 1 and 5 each had one gene (Figure 2). Furthermore, the majority of (70.22% (415)) *S. caninervis* TF genes were disrupted by introns, which varied from 1 to 17, while the remaining 29.78% (176) TF genes were intron-less (Table S1). Introns play a functional role in the regulation of gene expression. Exon shuffling involves the formation of a new gene during the plant evolution process [31].

### 2.3. GO Term, KEGG Pathway, and Cis-Element Analysis of Tfs in S. caninervis

**Figure 3:**
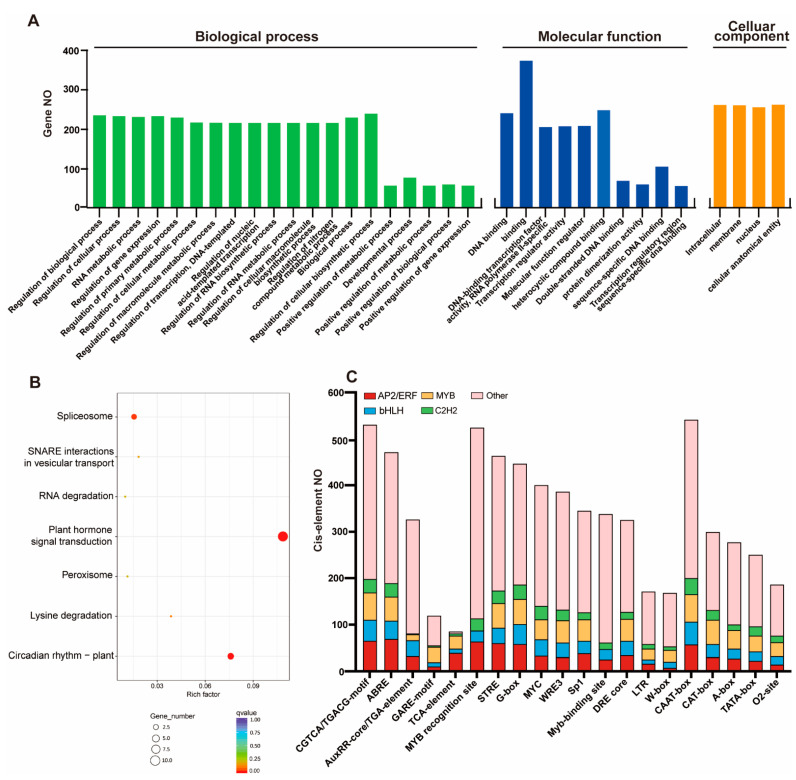
Molecular functional analysis of TF families in *S. caninervis*. (**A**) Gene ontology (GO) analysis of TF protein members. (**B**) KEGG pathway analysis of TF members. (**C**) Prediction of *cis*-element analysis of TF gene promoter regions.

To investigate the potential regulatory functions of 591 TFs in *S. caninervis*, GOterm was used. *ScTF* proteins were divided into three subgroups by GO annotation based on their classifications as a biological process, cellular component, and molecular function. GO results assigned *ScTF*s into 20 groups of biological processes. Several TF genes were involved in the regulation of transcription, cellular macromolecule biosynthetic process, gene expression, nitrogen compound metabolic process, primary metabolic process and DNA-templated (Figure 3A). In the cellular component, TF genes were relatively enriched in the nucleus, membrane, intracellular, and cellular anatomical entity. In the molecular function prediction of TF genes, they were theoretically associated with binding activities and transcription regulator activities (Figure 3A). The KEGG pathway analysis showed that the majority of TF members were associated with plant hormone signal transduction, circadian rhythm–plant, spliceosome, and lysine degradation (Figure 3B).

TFs can bind to specific DNA-sequences in the promoters of target genes and regulate their expression patterns under various environmental conditions [32,33]. In *S. caninervis*, the promoters of the TF genes contained various *cis*-elements such as CAAT-box, MYB-recognition site, as-1, G-box, STRE, CGTCA-motif, ABRE, and MYC (Figure 3C). In this investigation, many AP2/ERFs and C2H2-zinc finger promoter regions had ABRE and CGTCA/TGACG-motif elements (Table S5). The CGTCA-motif (*cis*-acting regulatory element involved in the MeJA-responsiveness) and CAAT-box (common *cis*-acting element in promoter and enhancer regions) were found in the promoter sites of all *S. caninervis* MYB genes (Figure 3C and Table S4). G-box and CAAT-box were detected on all bHLH promoter sites. Several *cis*-regulatory elements associated with hormone responses such as CGTCA-motif, ABRE, GARE-motif, TGA-element, AuxRR-core, and TCA-element, were identified in the promoter regions of AP2/ERF, MYB, and bHLH TFs (Figure 3C). Furthermore, some AP2/ERF, MYB, and bHLH promoter regions have a mixture of different hormone-related *cis*-regulatory elements. The result also identified other *cis*-regulatory elements related to diverse stresses such as G-box, STRE, Sp1, DRE core, I-box, MBS, MYC, GATT-motif, TCCC-motif and LTR (Figure 3C and Table S4).

### 2.4. Evolutionary Conservation of the Top Four TF Families in S. caninervis

**Figure 4:**
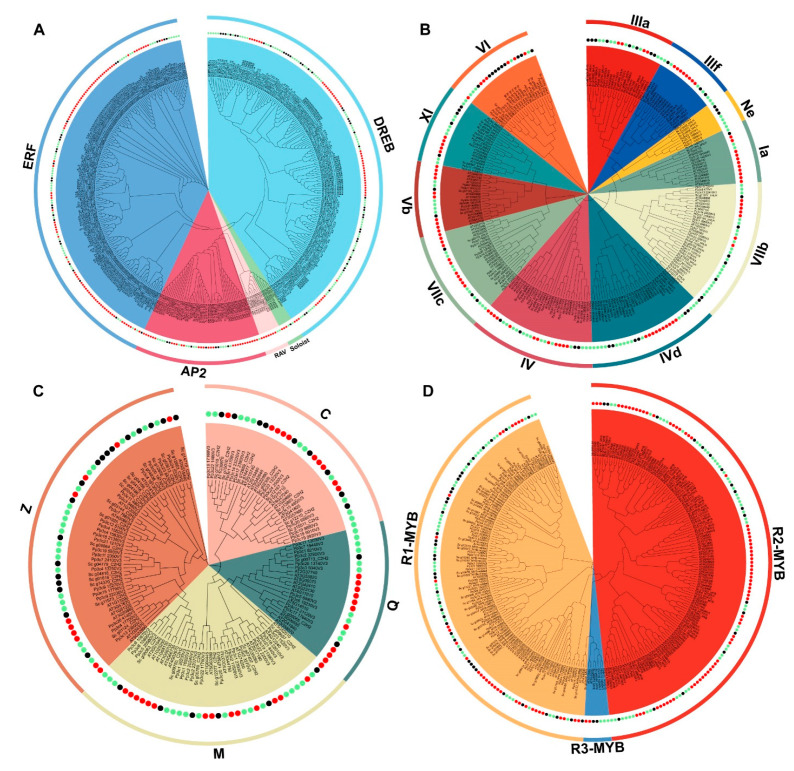
Molecular evolution analysis of the top four TF families in *S. caninervis*. Phylogenetic tree relationships of (**A**) AP2/ERF proteins. (**B**) bHLH proteins. (**C**) C2H2-zinc finger proteins. (**D**) MYB proteins in *S. caninervis*, *A. thaliana*, and *P. patens*. Black, green, and red dots mark *S. caninervis*, *P. paten*, and *A. thaliana*, respectively.

To study the molecular evolutionary relationships of AP2/ERF, MYB, bHLH, and C2H2-zinc finger proteins in *S. caninervis*, Arabidopsis and *P. patens* TFs were used to generate phylogenetic tree analysis with the neighbor-joining (NJ) method. Based on unrooted phylogeny tree analysis, the AP2/ERF, MYB, bHLH, and C2H2-zinc finger members were divided into different subgroups (Figure 4). The results showed that there was an unequal representation of the TF proteins from the three species within the specific subgroup (Figure 4). Based on the tree topology and sequence similarities, the AP2/ERF TF family was assigned to the AP2, DREB, ERF, RAV, and soloist subfamilies (Figure 4A). To classify these *S. caninervis* subfamilies, previous annotations of the plant AP2/ERF subfamilies have been considered [34,35]. The DREB subfamily had the most AP2/ERF members (*S. caninervis* (41), *A. thaliana* (48), and *P. patens* (75) genes) followed by The ERF subfamily (*S. caninervis* (26), *A. thaliana* (84) and *P. patens* (38) genes), and AP2 subfamily (*S. caninervis* (7), *A. thaliana* (28) and *P. patens* (14) genes). However, the lowest number of AP2/ERF genes belonged to the RAV subfamily (*A. thaliana* (7) and *P. patens* (2) genes), and AP2/ERF genes were assigned to the soloist subfamily (*S. caninervis* (1), *A. thaliana* (3) and *P. patens* (2) genes) (Figure 4A). Eleven bHLH family subgroups were identified and designated as 1a-NE (Figure 4B). To annotate these *S. caninervis* bHLH subgroups, we consider the earlier annotation of the Arabidopsis bHLH subfamilies as proposed by Pires and Dolan [36]. Ten subgroups (Ia to IX) contained bHLH genes from three plant species, and the VIIb subgroup had the highest number of genes with 34, followed by IVd and IV with 30 and 29 genes, respectively (Figure 4B). Whereas the Ne subgroup contained the lowest members, with only nine genes from desert mosses (*S. caninervis* (three genes) and *P. patens* (six genes)), indicating that this subgroup may have losses in the vascular plant genome. The C2H2-zinc finger family was categorized into four main groups: Q, M, Z, and C (Figure 4C). The number of genes in the C2H2-zinc finger family varied by group. Group Z contained the largest number of genes (*S. caninervis* (18), *A. thaliana* (10), and *P. patens* (22) genes), followed by groups M (*S. caninervis* (6), *A. thaliana* (15) and *P. patens* (17)) and C (*S. caninervis* (9), *A. thaliana* (10) and *P. patens* (12) genes). On the other hand, the lowest numbers of C2H2-zinc finger genes were found in group Q (two *S. caninervis*, 10 *A. thaliana* and 10 *P. patens* genes). MYB TF family is divided into three major subfamilies including 1R-MYB *S. caninervis* (37), *A. thaliana* (67) and *P. patens* (47)), 2R-MYB *S. caninervis* (19), *A. thaliana* (33) and *P. patens* (44)) and 3R-MYB (*S. caninervis* (2), *A. thaliana* (2) and *P. patens* (3)) (Figure 4D). MYB protein domains were specific to distinctive subfamilies, indicative of a particular role of these subgroups. Our results suggest that 1R-MYB is functionally important and evolutionarily conserved in *S. caninervis*. Importantly, several TF gene homologous groups were gathered by plant species within a particular subgroup, which potentially referred to that plant species (Figure 4).

### 2.5. Protein-Protein Interactions Analysis of the Top Four TF Families

**Figure 5:**
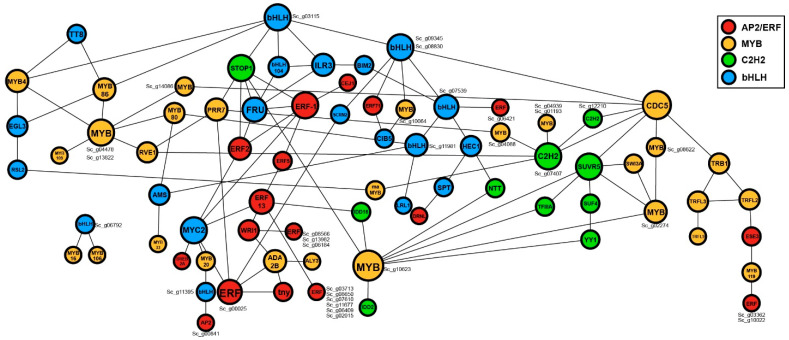
Protein-protein interaction networks of four overrepresented transcription factor families (AP2/ERF, MYB, bHLH, and C2H2) in *S. caninervis*. Different node colors indicate different TF family genes, and each edge indicates an interaction between two proteins. Red, yellow, green, and blue nodes mark AP2/ERF, MYB, C2H2, and bHLH TF families, respectively. The number of interacting genes was indicated by cycle size.

Protein-protein interaction is expressly important for gaining more insight into the functional roles of the proteins. To systematically examine the protein network interactions of *S. caninervis* TFs, we selected four overrepresented TF families (AP2/ERF, MYB, bHLH, and C2H2-zinc finger). The results found that 169 TFs (63 AP2/ERF, 49 MYB, 37 bHLH, and 20 C2H2-zinc finger members) potentially interacted according to their closer homologs in Arabidopsis (Figure 5 and Table S5). Apart from their regulatory functions in a specific pathway, the majority of TFs (AP2/ERF, MYB, bHLH, and C2H2-zinc finger) exhibited interaction network characteristics (Figure 5) allowing them to be involved in a wide variety of abiotic stress tolerance [37,38,39]. In this network, many TFs interact with multiple TFs that are not only in the same family but also from different families. ERF-1 (*Sc_g15827_ERF*, *Sc_g15828_ERF*, *Sc_g00099_ERF*, *Sc_g04891_ERF*, *Sc_g08334_ERF*, *Sc_g14120_ERF* and *Sc_g00444_ERF*) physically interacted with EFR-2 (*Sc_g04542_ERF*), CEJ1 (*Sc_g02134_ERF*), EFR-5 (*Sc_g05418_ERF*), FRU (*Sc_g00742_bHLH*), MYC2 (*Sc_g00589_bHLH*, *Sc_g00591_bHLH* and *Sc_g00606_bHLH*) and STOP1 (*Sc_g01124_C2H2*) (Figure 5 and Table S5). MYC2 potentially interacted with five members of ERFs (EFR-1, EFR-5, EFR-13, *Sc_g00025_ERF* and DREB2A (*Sc_g02107_ERF*) and MYB20 (*Sc_g13885_MYB*). bHLH_AT4G37850 (*Sc_g03115_bHLH*) physically interacted with bHLH_*AT1G68920* (*Sc_g09345_bHLH* and *Sc_g08830_bHLH*), bHLH104 (*Sc_g01930_bHLH*), ILR3 (*Sc_g01928_bHLH*), STOP1 (*Sc_g01124_C2H2*), MYB4 (*Sc_g15787_MYB* and *Sc_g00747_MYB*) and MYB86 (*Sc_g11316_MYB* and *Sc_g14329_MYB*) (Figure 5 and Table S1). CDC5 (*Sc_g04917_MYB*) importantly interacted with MYB-related_*AT2G47210* (*Sc_g08622_MYB-related*), TRB1 (*Sc_g00407_MYB-related*), *AT3G11450* (*Sc_g14086_MYB-related*), SUVR5 (*Sc_g15511_C2H2*), *AT5G26610* (*Sc_g12210_C2H2*), MYB-related_AT1G74250 (*Sc_g07407_C2H2*) and bHLH_*AT1G68920* (*Sc_g09345_bHLH* and *Sc_g08830_bHLH*). STOP1 (*Sc_g01124_C2H2*) probably interacted with two ERF members (ERF-1, ERF-2), two bHLH genes (bHLH_*AT4G37850* (*Sc_g03115_bHLH*) and FRU (*Sc_g00742_bHLH*) and one member of MYB (PRR7 (*Sc_g07847_MYB*).

### 2.6. MicroRNAs and Their Target Transcription Factors

**Figure 6:**
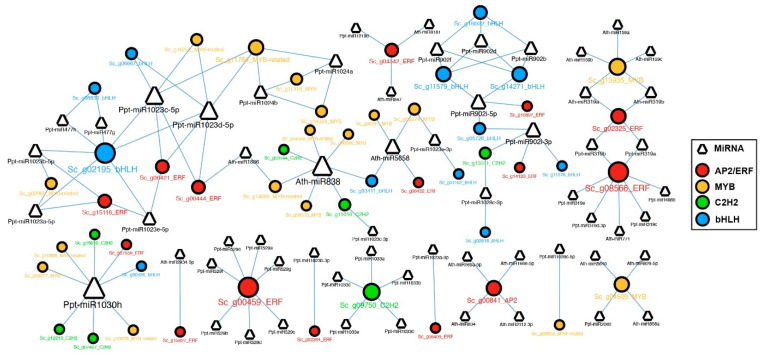
MicroRNA targeted four overrepresented TFs in *S. caninervis*. Cycle nodes indicate TFs and triangle nodes indicate microRNAs. Four TF family members were represented by different colors. Cycle and triangle sizes reflect the numbers of TFs or microRNAs.

To investigate the molecular function of TF genes in *S. caninervis*, we used predictive miRNAs (http://plantgrn.noble.org/psRNATarget) (accessed on 1 February 2023) [40], PmiREN (https://www.pmiren.com) (accessed on 1 February 2023) [41] and target genes (http://bioinformatics.psb.ugent.be/webtools/tapir/) (accessed on 1 February 2023) to examine if some TF genes are targeted by miRNAs. The result found that a total of *S. caninervis* 46 TFs were identified as being targeted by 43 miRNAs (eight miRNAs from *Arabidopsis* and 35 miRNAs from *P. patens*) (Figure 6). Several TFs were targeted by at least one miRNA (Figure 6); for example, *Sc*_*g09750_C2H2* was targeted by five miRNAs (*Ppt*-*miR1033a*,b,c,d,e), *Sc*_*g04509_MYB* targeted by four miRNAs (*Ath*-*miR5640*, *Ath*-*miR829-5p*, *Ath*-*miR858a*, and *Ppt*-*miR2082*) and *Sc*_*g13935_MYB* targeted by five miRNAs (*Ath*-miR159a,b.c and *Ath*-miR319a,b). However, *Ath-miR1886* possibly targeted the *Sc_g00444_ERF* gene, and *Ath-miR5658* targeted four TF members (*Sc*_*g00747_MYB*, *Sc*_*g03411_bHLH*, *Sc_g06422_ERF* and *Sc_g02274_MYB*) (Figure 6 and Table S6).

### 2.7. Expression Profile Analysis of Four Overrepresented TFs under Chilling and Freezing Stresses Based on Transcriptome Data

**Figure 7:**
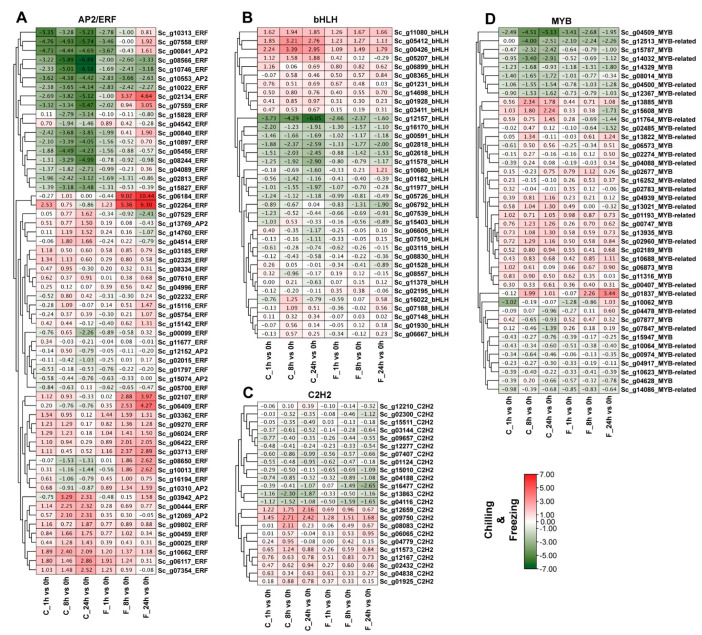
Expression analysis of the top four TF family genes was examined using hierarchical clustering in different time-series of chilling and freezing stresses. FPKM values of representative transcript levels of TF members. The standardized expression data was used to generate a heatmap with hierarchical clustering based on the Manhattan correlation with average linkage using the MeV software package. Expression levels of (**A**) AP2/ERF, (**B**) bHLH, (**C**) MYB, (**D**) C2H2-zinc finger genes under chilling and freezing stresses at 1, 8, and 24 h. The heatmap shows the change in the average log2 (fold-change) of each transcript.

Transcriptome results showed that the majority of the Sc*AP2/ERF*, *MYB*, *bHLH*, and *C2H2* genes in *S. caninervis* are responsive to cold stress (chilling and freezing). A total of 166 TFs from 222 Sc*AP2/ERF*, *MYB*, *bHLH*, and *C2H2* genes were significantly changed in transcript abundances under cold stress at 1, 8, and 24 h as compared to the control (0 h) (Figure 7A–D). A significant number (60/75) of AP2/ERF (7 AP2 and 53 ERFs) genes were induced by chilling and freezing stresses at different time-series in comparison with the control (Figure 7A), suggesting that these genes might play an important role in cold responses. Under freezing stress, the transcript performances of *Sc_g02134_ERF*, *Sc_g06184_ERF*, and *Sc_g02264_ERF* were significantly up-regulated at 8 and 24 h. However, *Sc_g10553_AP2*, *Sc_g08566_ERF*, *Sc_g10022_ERF*, and *Sc_g10746_ERF* were significantly down-regulated during cold exposure in the stressed *S. caninervis*, implying their functional roles in adaptation mechanisms to cold conditions (Figure 7A). Under cold exposure, a total of 36 (36/52) bHLH genes were differentially expressed in the stressed *S. caninervis* in comparison with the control (Figure 7B). *Sc_g00426_bHLH*, *Sc_g05412_bHLH*, and *Sc_g11080_bHLH* were significantly up-regulated while, *Sc_g12157_bHLH*, *Sc_g16170_bHLH Sc_g02618_bHLH*, and *Sc_g02818_bHLH* were down-regulated in the stressed *S. caninervis* by cold treatments at 1, 8 and 24 h (Figure 7B). The great number of differentially expressed MYB TFs (41/58) (17 MYB and 24 MYB-related) genes revealed low transcript levels in *S. caninervis* under cold conditions (Figure 7D). *Sc_g04509_MYB*, *Sc_g12513_MYB-related*, *Sc_g14329_MYB*, and *Sc_g04500_MYB-related* transcript abundances were down-regulated in response to cold stress (Figure 7D). The transcript levels of *Sc_g13885_MYB* and *Sc_g15608_MYB* were up-regulated under chilling stress by more than 1.5 fold-changes, and *Sc_g01837_MYB-related* was up-regulated under freezing stress at 8 and 24 h by more than 2.5 fold-changes (Figure 7D), suggesting that these genes might play an important role in regulating chilling and freezing stress tolerance. A total of 23 (23/35) C2H2-zinc finger genes were relatively induced in transcript abundances under chilling and freezing stresses at different time points (Figure 7C). *Sc_g09750_C2H2* and *Sc_g12659_C2H2* transcripts were up-regulated in response to *S. caninervis* cold stress tolerance, indicating that these transcription factors may also be associated with the regulation of cold stress tolerance.

### 2.8. Expression Profile Analysis of Four Overrepresented TFs under Dehydration-Rehydration Stress Based on Transcriptome Data

**Figure 8:**
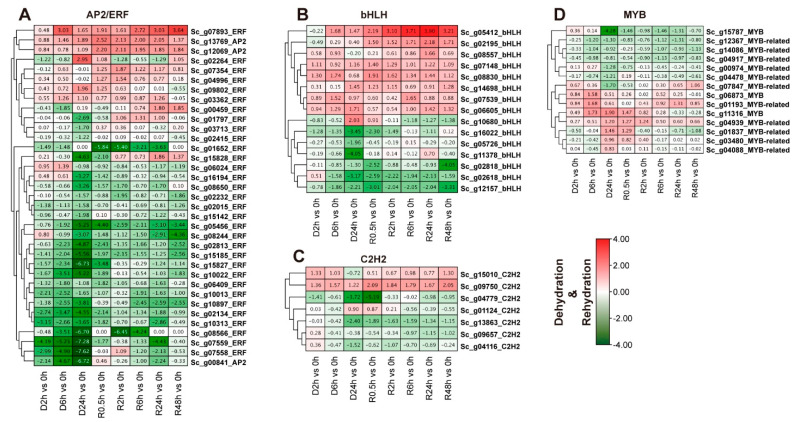
Expression profiles of the top four TF family genes were analyzed using hierarchical clustering in different time-series of dehydration-rehydration stress. FPKM values of representative transcript levels of TF members. The standardized expression data was used to generate a heatmap with hierarchical clustering based on the Manhattan correlation with average linkage using the MeV software package. Expression levels of (**A**) AP2/ERF, (**B**) bHLH, (**C**) MYB, (**D**), C2H2-zinc finger genes under dehydration stress at 2, 6, and 24 h, and rehydration conditions at 0.5, 2, 6, 24 and 48 h. The color scale below the heatmap represents the expression pattern; green shows down-regulated and red shows up-regulated genes. The heatmap shows the change of the average log2 (fold-change) of each transcript.

The transcript abundance changes of four overrepresented *ScTF* families were further investigated in response to dehydration and rehydration stresses. In Figure 7, only 71 TFs out of 220 had significantly different transcript abundances at various times of D-R treatment in *S. caninervis* as compared to the control (0 h). Approximately half (35/76) of the AP2/ERF (3 AP2 and 32 ERF TFs) genes were differentially expressed in *S. caninervis* response to D-R stress (Figure 8A). The transcript abundances of *Sc_g02813-ERF*, *Sc_g05456_ERF*, *Sc_g15827_ERF*, *Sc_g00841_ERF*, *Sc_g02134_ERF*, *Sc_g07559_ERF*, *Sc_g10013_ERF*, *Sc_g07559_ERF*, and *Sc_g15185_ERF* were significantly down-regulated in the treated *S. caninervis* (Figure 8B). On the other hand, *Sc_g07893_ERF*, *Sc_g13769_AP2*, and *Sc_g12069_AP2* were up-regulated in the treated *S. caninervis* at different time-series of the D-R process as compared to the control (Figure 8A). The bHLH family has 15 differentially expressed genes, the majority of these genes were up-regulated in response to D-R stress (Figure 8B). For example, the transcript levels of the *Sc_g05412_bHLH*, *Sc_g08830_bHLH*, *Sc_g06605_bHLH*, *Sc_g14698_bHLH*, and *Sc_g07539_bHLH* genes were relatively increased under D-R stress. *Sc_g12157_bHLH* and *Sc_g02818_bHLH* transcripts were down-regulated in the treated *S. caninervis* by desiccation stress. Within the MYB family, 14 MYB (3 MYB and 11 MYB-related TFs) genes were responsive to D-R stress (Figure 8B). The transcript abundances of *Sc_g15787_MYB*, *Sc_g04917_MYB-related*, and *Sc_g14086_MYB-related* were down-regulated, but *Sc_g04939_MYB_related* and *Sc_g01193_MYB_related* were up-regulated in stressed *S. caninervis* under D-R condition. In total, the transcript abundance of seven C2H2-zinc finger genes varied relatively in abundance patterns depending on the D-R condition (Figure 8C). (*Sc_g09750_C2H2* and *Sc_g15010_C2H2* were up-regulated, while *Sc_g04779_C2H2* and *Sc_g_1363_C2H2* were down-regulated in stressed *S. caninervis* by rehydration and dehydration at 2 and 6 h (Figure 8D).

### 2.9. Validation of TFs Transcript Abundance by RT-qPCR

**Figure 9:**
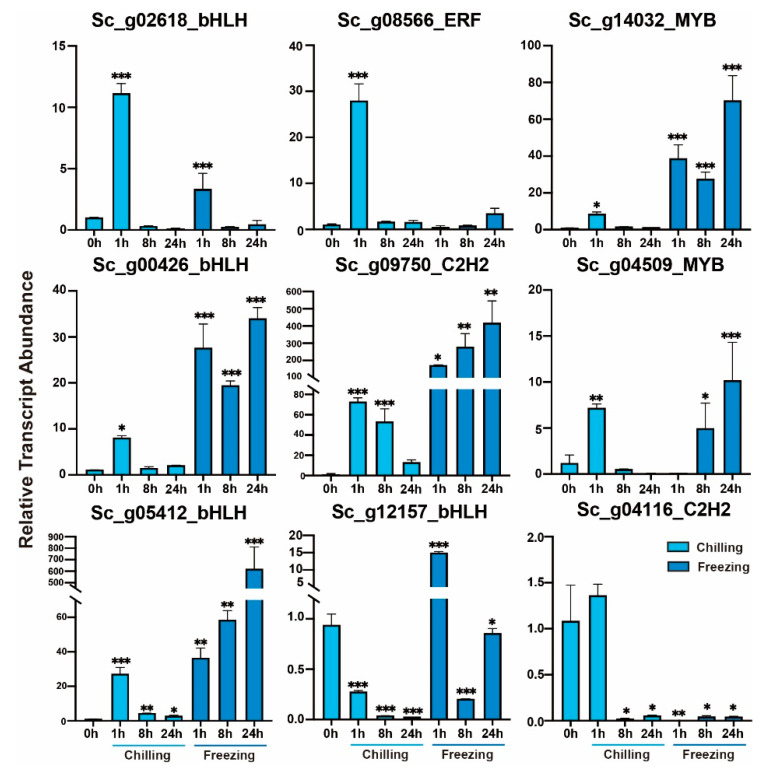
The relative transcript abundance analysis of nine represented TFs in response to chilling (4 °C) and freezing (−4 °C) stresses by RT-qPCR at 0, 1, 8, and 24 h. Data are represented as means ± SD of three independent biological replicates of each treatment. The significant difference compared with the control (0 h), LSD multiple comparison tests: * *p* < 0.05, ** *p* < 0.01, ***, *p* < 0.001.

To further explore the functional roles of these TF families in response to cold and desiccation stresses, we selected nine TF genes including, four *bHLH*, two *MYB*, two *C2H2*-zinc fingers, and one *AP2/ERF*, with different transcript abundances for verification by RT-qPCR assay (Figure 9). All of the selected TF genes showed a significant change in transcript abundance at different time-series of chilling and freezing conditions, indicating that these TFs may be involved in the *S. caninervis* cold stress response. The transcript abundance of *Sc_g02618_bHLH* and *Sc_g08556_ERF* was significantly elevated to about 10-fold and 25-fold at 1 h under chilling stress, while their expression patterns were effectively induced by freezing stress (Figure 9). The transcript abundances of *Sc_g14032_MYB*, *Sc_g00426-bHLH*, *Sc_g09750_C2H2*, *Sc_g04509_MYB*, and *Sc_g05412_bHLH* were significantly increased at various times of cold stress but their relative expression patterns under freezing stress were higher than those under chilling stress. Among them, the transcript levels of *Sc_g09750_C2H2* and *Sc_g05412_bHLH* were dramatically increased to about 500-fold at 24 h under freezing stress (Figure 9). The transcript level of *Sc_g12157_bHLH* was significantly decreased under chilling stress, whereas it was significantly increased with freezing stress at 1 h. *Sc_g04116_C2H2* was highly increased with the influence of chilling at 1h and significantly decreased with chilling stress at 8 and 24 h and freezing stress (Figure 9).

**Figure 10:**
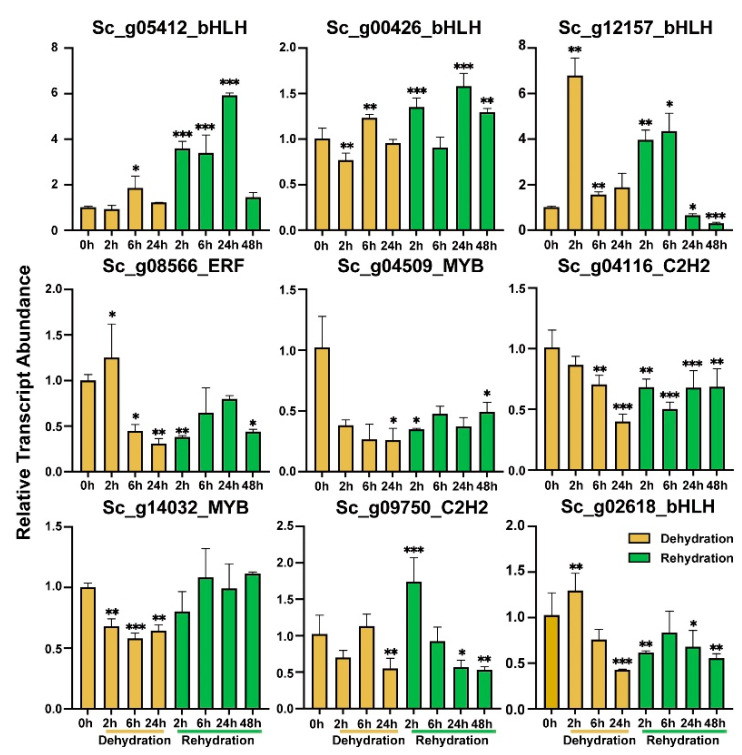
The relative transcript abundance analysis of nine represented TFs in response to dehydration (2, 6, and 24 h) and rehydration (2, 6, 24, and 48 h) stresses by RT-qPCR. Data are represented as means ± SD of three independent biological replicates of each treatment. The significant difference compared with the control (0 h), LSD multiple comparison tests: * *p* < 0.05, ** *p* < 0.01, ***, *p* < 0.001.

The relative transcript abundance of the nine selected TFs under dehydration stress and rehydration conditions at different time points was also examined by RT-qPCR (Figure 10). All tested *S. caninervis* TFs responded to the D-R condition (Figure 10). The transcript abundance of three *ScbHLH* genes (*Sc_g05412_bHLH* and *Sc_g00426_bHLH)* was increased in stressed *S. caninervis* under dehydration at 6 and 24 h, and rehydration conditions (Figure 10). *Sc_g12157_bHLH* transcript abundance increased during D-R stress (2 and 6 h) but it was gradually reduced in response to rehydration conditions at 24 and 48 h. The transcript abundance of *Sc_g08556_ERF* and *Sc_g02618_bHLH* was significantly elevated at 2 h under dehydration stress but dramatically reduced with dehydration (6 and 24 h) and rehydration conditions. The transcript abundance of *Sc_g04509_MYB* and *Sc_g04116_C2H2* was significantly reduced with dehydration and rehydration stresses (Figure 10). The transcript abundance of *Sc_g14032_MYB* significantly decreased in response to dehydration stress but did not change significantly during the rehydration process. The *Sc_g09750_C2H2* transcript pattern was significantly increased at 2 h of dehydration stress and decreased in response to dehydration stress at 24 h and dehydration process at 24 and 48 h (Figure 10).

## 3. Discussion

### 3.1. Genome-Wide Identification and Comparative Analysis of TFs

TFs can recognize and bind to a specific sequence of DNA-binding and interact with various proteins in the transcriptional process to regulate the expression pattern of a great number of genes that are involved in plant growth and survival strategies under different stress conditions [42,43]. In this study, the identification and characterization of TF members in *S. caninervis* based on the genome were completed. We analyzed the TFs that existed in *S. caninervis* and analyzed how TF functions and molecular roles might be explained in this unique model plant. More remarkably, we determined protein domain specificity for diverse TF members related to various families (Figure 1 and Figure S1) for which there was little information available in *S. caninervis*. Finally, 591 TF members were identified in *S. caninervis* and distributed among 52 TF families. The percentage of *S. caninervis* TF members (3.6%) was significantly lower than that in vascular plants, which ranged from 6% to 10% of the total genes [10]. The variation in TF numbers among different plants provided a stronger sign of involvement in specific functional roles during plant growth, development, and response to diverse stresses. The complication of multi-cellular organism evolution events is associated with an increase in the number of TF member families and regulatory protein expansion [44]. Meanwhile, the identified *S. caninervis* TF numbers were significantly lower than those in *P. patens*, this may be partially explained by the fact that *S. caninervis* has experienced a single whole genome duplication (WGD) [29], whereas, *P. patens* has undergone two WGD events [45]. The AP2/ERF family is one of the largest TF families in moss species in comparison with algae and higher plants (Figure 1). In *S. caninervis*, AP2/ERF family is divided into four subfamilies including AP2, ERF, DREB, and soloist, and the absence of RAV genes. Furthermore, the ratio of the ERF subfamily is relatively increased, while the proportion of theAP2 subfamily is reduced in *S. caninervis* as compared with *A. thaliana*, *P. trichocarpa*, *O. sativa*, and *Z. mays* (Table S2). This result is in agreement with our previous reports [46]. The MYB family is the second largest TF group in *S. caninervis* and is classified into three distinct subgroups including MYB-R1, MYB-R2, and MYB-R3, and lacks the MYB-R subgroup. The proportion of MYB-R1 is relatively larger in *S. caninervis* as compared to *V. carteri*, *P. patens*, *O. sativa*, *Z. mays*, *A. thaliana*, upland cotton, and *B. napus* (Table S3), while, the majority of MYB genes are related to the MYB-R2 subgroup in higher plants [30]. MYB protein members within the same subgroups had the same protein domain in terms of R repeat and distribution, which suggested that the MYB TF family within a specific subfamily, could share a similar molecular function (Table S3). This result was in agreement with a prior report that concluded that members of the MYB TF family with similar protein compositions were classified into the same subgroup [47]. bHLH TFs are characterized by a basic helix–loop–helix domain with two parts, a basic DNA binding domain at the N-terminal and a protein interaction domain at the C-terminal [44]. Previous results showed that bHLH TFs in several plant species were gradually increased from algae to plants in association with increased plant adaptation complexities [48]. In the *S. caninervis* genome, the bHLH family is the third-largest TF member, which can be divided into 11 groups based on evolutionary conservation. According to protein domain analysis, C2H2-zinc finger TFs in *S. caninervis* were classified into four major subgroups including Q-type, M-type, Z-type, and C-type (Figure 4C). The majority of C2H2-zinc finger genes in this study belonged to groups Z and C, in contrast to earlier findings in higher plants [49].

### 3.2. The Functional Prediction of the Top Four TF Families by Comprehensive Analysis

The functional roles of most TFs remain largely unknown; therefore, we used GO analysis and the KEGG pathway to investigate the biological roles of TFs in the regulation of molecular responses in *S. caninervis*. Bioinformatics analysis revealed that TFs were potentially associated with the regulation of transcription, DNA-templated, RNA process, gene expression, binding activities, plant hormone signal transduction, circadian rhythm–plant, and spliceosome (Figure 3A). These results indicated that *S. caninervis* TF genes may play important roles in the regulation of gene expression and various hormone signaling pathways in plant cell responses to changes in environmental stimuli. In this investigation, we observed a strong overrepresentation of ABRE and CGTCA-motif/TGACG-motif elements in the promoter regions of AP2/ERFs and C2H2-zinc finger TF families (Table S5), suggesting that ABA and JA might be involved in the regulation of stress responses in *S. caninervis* [50]. Meanwhile, several AP2/ERF, MYB, and bHLH promoter regions have a mixture of diverse hormones-related *cis*-regulatory elements (Figure 3B and Table S5), supporting the functional role of TFs in the regulation of various hormone signaling pathways [51,52]. AP2/ERFs are regulated by hormone pathways and modulate hormone signaling and biosynthesis in plants [53]. The outcome of this examination showed that several *cis*-regulatory elements related to TFs could be involved in regulating abiotic stress responses in *S. caninervis*.

TFs interact with other proteins in the regulation of transcriptional processes [54]. The results of protein-protein interactions found that the majority of *ScTF*s physically interacted within the family as well as with other TF families (Figure 5). In plants, AP2/ERF, bHLH, and MYB are essential abiotic stress-related TF families [51,55]. ERF1 is a key module in ethylene signaling, playing an important function in stress responses and participating in the delay in flowering time in Arabidopsis [56,57]. In plant-pathogen interactions, ERF1 physically integrates ethylene and JA signaling to trigger the expression levels of defense-related genes [58]. It was found that ERF1 probably targets several stress-related genes in response to various abiotic stress [59]. MYC2 is associated with the regulation of abiotic stress responses such as drought and low temperatures [60]. In this study, *ScERF-1* and *ScMYC2* have the greatest number of potential interacting proteins with high similarity. *ScERF-1* could interact with six TFs belonging to three TF families including, AP2/ERF (EFR-2, CEJ1, and EFR-5), bHLH (FRU and MYC2), and C2H2-zinc finger (STOP1), and these TF members also interacted with other TF families. *ScMYC2* potentially interacted with five members of ERFs (EFR-1, EFR-5, EFR-13, *Sc_g00025_ERF*, and DREB2A) and one MYB gene; our results suggest that *ScERF1* and *ScMYC2* play key roles in the abiotic stress regulatory network of *S. caninervis*. Importantly, TFs and miRNAs can regulate each other [61], and they are involved in plant stress responses [62]. In the model plants, microRNA159 (*Ath*-*miR159*a,b,c) is a highly preserved miRNA in plants and contributes to stress responses [63]. Ath-*miR5658*, *Ath*-*miR858*, and *Ath*-*miR1886* were also found to be responses to various stress tolerances [64,65,66]. In this work, we identified several miRNAs from Arabidopsis and *P. patens* that theoretically target overrepresented TF families in *S. caninervis*, similar to previous report*s*, stress-related miRNAs also target many TFs in *S. caninervis*, such as *Ath-miR5658* potentially targeted four *ScTF*s members (Sc_*g03411_bHLH*, *Sc_g06422_ERF*, and *Sc_g02274_MYB*) and *Ath-miR159* can specifically target *Sc*_*g13935_MYB.* Meanwhile, one TF can be regulated by many miRNAs, such as *Sc_g00444_ERF* which was targeted by three miRNAs and *Sc_g04509_MYB* was targeted by four miRNAs, implying that miRNAs might contribute to abiotic stress response regulatory network interactions in *S. caninervis*. Further research on the network interaction between TFs and miRNAs in *S. caninervis* will contribute significantly to elucidating their biological functions in abiotic stress responses.

### 3.3. Transcript Abundances of AP2/ERF, MYB, bHLH, and C2H2-Zinc Finger Genes Associated with Cold and Dehydration-Rehydration Stresses

The comparative analysis of single TF families indicates that transcriptional regulators are frequently specialized in specific responses to environmental stimuli [26,67,68]. Recently, extensive studies have elucidated the regulatory functions played by AP2/ERF, MYB, bHLH, and other TFs in responses to biotic and abiotic stresses in plants [48]. The majority of transcripts encoding AP2/ERF, MYB, bHLH, and C2H2 TFs were induced by both cold and D-R stress, indicating that cold and desiccation responses may share common signal transduction events. Specifically, the result also found that there are transcript TFs strongly induced by cold stress (specifically freezing) rather than D-R, such as the transcript patterns of *Sc_g09750_C2H2* and *Sc_g05412_bHLH* dramatically increased about 500-fold at 24 h during freezing stress but only increased 6-fold during D-R stress. Some of the TFs were only induced by cold and not altered by D-R stress (Figure 9 and Figure 10), such as the transcript patterns of *Sc_g06184_ERF* was significantly up-regulated under freezing stress at 24 h with more than 1000 fold, while it was not induced during the D-R process (Figure 7 and Figure 8), suggesting that this gene specifically plays regulatory role in *S. caninervis* response to freezing stress.

Previous studies have shown that AP2/ERF members play an important role in the regulation of abiotic stress responses [69]. In the model moss *P. patens*, AP2/ERFs are associated with the regulation of dehydration-rehydration, light [70], and cold stress responses [71]. *ScDREB* genes in *S. caninervis* significantly respond to multiple stresses and notably enhance drought, salt, and cold stress tolerance in transgenic yeast and Arabidopsis [26], suggesting that the AP2/ERF genes play important roles in moss stress responses. In this study, among the four over-represented TF families, the AP2/ERF family was the key differentially expressed TF in *S. caninervis* with great signal induction and detection under both cold and D-R treatments, indicating that AP2/ERF TF family may significantly contribute to cold and desiccation stress responses. It was reported that several members of bHLH TFs are involved in the regulation of abiotic stresses, such as light [72], cold [73], drought [74], salt [75], and hormonal signals [76]. The majority of detectable *bHLH* genes were increased in stressed *S. caninervis* by cold and dehydration-rehydration processes (Figure 7 and Figure 8). RNA-seq and RT-qPCR results showed that the transcript abundances of bHLH members were strongly increased by dehydration-rehydration stress, indicating that these genes may play functional roles in responses to abiotic stress tolerance in *S. caninervis*. The induced abundance of transcripts encoded by specific TFs could be reflected in the accumulation of mRNAs, which are associated with regulating the increasing abundance of cryoprotectant compounds during cold exposure [48] and dehydration responses [77]. The different expression patterns of these TFs between cold stress and desiccation stress indicated that in *S. caninervis*, these TFs might have multiple roles in replying to various abiotic stresses. The research findings highlight AP2/ERFs genes as important stress regulators in *S. caninervis* [20,48].

## 4. Material and Methods

### 4.1. Identification and Genetic Mapping of S. caninervis TFs

To identify the features of transcription factor families in *S. caninervis*, blast tools were used to search against the *S. caninervis* genome database. *S. caninervis* protein sequences were obtained from the National Center for Biotechnology Information (https://www.ncbi.nlm.nih.gov/genome/97510) (accessed on 1 February 2023) for systematic analysis. A local BLASTP using corresponding protein sequences of TF families was obtained from the plant TFs database (http://planttfdb.cbi.edu.cn/) (accessed on 1 February 2023) and National Center for Biotechnology Information (https://www.ncbi.nlm.nih.gov/Structure/cdd/wrpsb.cgi) (accessed on 1 February 2023). The Hidden Markov Model (HMM) profile of the TF domains was downloaded from the Pfam database (http://pfam-legacy.xfam.org) (accessed on 1 February 2023) [78]. Both methods of BLASTP were used to search against the *S. caninervis* proteomic database, with *E*-value ≤ 10^−10^. All the identified transcription factor sequences were systematically aligned using ClustalW alignment (https://www.ebi.ac.uk/Tools/msa/clustalw2/) (accessed on 1 February 2023) to remove the redundant sequences. Additionally, the SMART database (Simple Modular Architecture Research Tool: http://smart.embl-heidelberg.de/) (accessed on 1 February 2023) and InterPro online (http://www.ebi.ac.uk/interpro/) (accessed on 1 February 2023) were used to validate the presence of TF domains in *S. caninervis* and only protein sequences with known TF domains were considered for further investigation. The accuracy of TF prediction and identification was improved by setting up two rules to identify and categorize TFs into their exact TF families. First, if a protein sequence in a family or superfamily had numerous TF domains, we categorized the TFs according to the specific domain of the protein family. The second rule is that if a TF protein sequence with multiple DNA-binding domains, we classify it into the specific family with the smallest *E*-value in DNA-binding domain prediction. The chromosomal locations of all *S. caninervis* TF members were identified through BLASTN searches against the *S. caninervis* genome database. Map gene 2chromosome v2 (http://mg2c.iask.in/mg2c_v2.0/) (accessed on 1 February 2023) online software was used to map *S. caninervis* TFs into chromosomes. The input files were prepared and contained the following detailed information: *S. caninervis* gene ID, start and end positions of TFs, chromosome ID of TF members, and corresponding chromosomal length.

### 4.2. Bioinformatics Analyses of S. Caninervis TF Families

Gene Ontology (GO) enrichment analysis of TFs was implemented by the GOseq R package. GO terms with a corrected *p*-value less than 0.05 were considered significantly enriched. We used KOBAS software to test the statistical enrichment of *S. caninervis* TFs in KEGG pathways with a corrected *p*-value less than 0.05 was considered significantly enriched. *Cis*-acting regulatory elements in the promoter of each TF gene sequence (1.5 kb upstream of the translation starting region) were conducted using the PlantCARE database (http://bioinformatics.psb.ugent.be/webtools/plantcare/html/) (accessed on 1 February 2023). To investigate the evolutionary conservation of the *S. caninervis* top four TF families, multiple sequence alignments of *S. caninervis*, Arabidopsis, and *P. patens* TF members were conducted using ClustalW (http://www.clustal.org/clustal2/) (accessed on 1 February 2023). Phylogenetic tree analysis was implemented by MEGA 6.0 software (http://www.megasoftware.net/) (accessed on 1 February 2023) using the NJ (neighbor-joining) method with 1000 bootstrap repetitions. The phylogeny tree was completed with the following standards: Substitution, Poisson Model, data subset to use, the p-distance, pairwise deletion, and replication. Furthermore, to get more insight into the regulatory functions of four overrepresented TFs (AP2/ERF, MYB, bHLH, and C2H2-zinc finger) in the molecular mechanisms underlying visa protein-protein interaction in *S. caninervis*, the STRING database (https://string-db.org) (accessed on 1 February 2023) was applied to perform the protein interaction networks [79]. We submitted *S. caninervis* TF sequences to the STRING database to predict the protein-protein interaction based on their closer Arabidopsis protein sequences (gene similarities greater than 50% with an *E*-value < 10^−20^). In order to predict miRNAs and their TF targets, several algorithm tools were used to identify miRNAs (http://plantgrn.noble.org/psRNATarget) (accessed on 1 February 2023) [40], PmiREN (https://www.pmiren.com) (accessed on 1 February 2023) [41], and TAPIR: Target prediction for plant microRNAs (http://bioinformatics.psb.ugent.be/webtools/tapir/) (accessed on 1 February 2023).

### 4.3. Expression Profiling of TFs in Stressed S. caninervis by Abiotic Stress

*S. caninervis* gametophytes were harvested from the Gurbantunggut Desert of Xinjiang Uygur Autonomous Region of China [31,32,33,34,35]. The *S. caninervis* sample was placed in Petri dishes and stored in an air-dried place at room temperature for at least one week, and then the gametophytes were fully rehydrated for 24 h before stress treatment. For cold stress (chilling and freezing), hydrated gametophores were gently and slowly dried with filter paper to remove the surface water, and evenly placed in the Petri dishes and treated with chilling (4 °C) and freezing (−4 °C) stresses in an artificial climate box (LRX-580A-LED, PRANDT, Ningbo, China), using 16/8 h light/dark photo-cycles (approximately 100 μmol m^−2^s^−1^ constant light, 50% relative humidity) as compared to the control (*S. caninervis* grown under the normal condition at 25 °C). *S. caninervis* samples were collected at 0 (control), 1, 8, and 24-h of cold exposure (chilling and freezing stress). The dehydration (slow drying) treatment was completed by placing the gametophytes in small wire baskets over saturated sodium nitrite solution (67% relative humidity at 25 °C) in a sealed glass desiccator reserved in the same incubator [80] and samples were collected at 0, 2, 6, and 24-h. Rehydration treatment of *S. caninervis* samples was reached by placing the dehydrated gametophytes after slow drying in a new Petri dish in the incubator at 25 °C in the light and adding distilled water but not submerging moss plants to fully hydrated gametophytes, and samples were harvested at 0, 0.5, 2, 6, 24, and 48-h. Three biological replicates for each sample were collected for the experimental procedures. Cold transcriptome data (chilling and freezing stress) and desiccation transcriptome data (dehydration and rehydration) are submitted to China National GeneBank DataBase (CNGBdb) under the accession numbers (CNP0003778) and (CNP0003370), respectively. To investigate the expression patterns of TFs in *S. caninervis* exposed to cold (chilling and freezing), dehydration, and rehydration stress treatments in comparison with the control (0-h). We calculated the expression patterns of each TF using Fragment per Kilobase of exon model per Million mapped reads (FPKM) with Cufflinks (Version 2.1.1) [81] (http://cufflinks.cbcb.umd.edu/) (accessed on 1 February 2023). Fold changes of various TFs expressed analysis and the related statistical computations of the two pairwise comparisons were conducted with the DESeq R package (1.10.1) [82]. Benjamini’s and Hochberg’s method was used to adjust the *p*-value by controlling the false rate [83]. Only transcription factor genes with corrected *p*-value ≤ 0.05 and |log2(Fold change)| > 1 were considered as differentially expressed TFs using TBtools [84].

RT-qPCR analysis was performed on CFX-96 Real-Time System (Bio-Rad, Hercules), using SYBR Premix Ex TaqTM II (TaKaRa) to validate gene expression patterns of RNA-seq. During chilling and freezing exposures, samples were harvested at 0, 1, 8, and 24 h. Under dehydration treatment, plant samples were collected at 0, 2, 6, and 24 h. For rehydration treatment, plant samples were collected at 0, 2, 6, 24, and 48 h, and all plant samples were completed with three biological replicates. The *ScTubulin* was used as a reference gene for the normalization of the relative expression level of each gene [85]. The relative quantification from three biological replicates was standardized to the reference gene and calculated by the 2^−ΔΔCt^ method [86].

## 5. Conclusions

Similar to other green plants, the desiccation stress-tolerant moss *S. caninervis* genome has significant numbers of TF families, but their ratio is less than that in higher plants. In this study, overrepresented TF families include the AP2/ERF, MYB, bHLH, and C2H2 genes, and the detailed categorization of each family is completed based on structural features. The combination of bioinformatics analysis and expression profiling of TFs in *S. caninervis* indicated that several members of the AP2/ERF and bHLH TFs might play a key role in the modulation of diverse pathways in response to abiotic stress tolerance. A comprehensive analysis of *S. caninervis* TFs and their evolutionary functions might help to elucidate the regulatory mechanism underlying abiotic stress responses in *S. caninervis*. Further research on functional validation of the key TFs and comparative studies with other plants will be crucial steps in understanding their molecular regulation and network interactions in response to various abiotic stresses.

## Data Availability

All related data are available within the manuscript and its additional files.

## Supplementary Materials

The following supporting information can be downloaded at: https://www.mdpi.com/article/10.3390/ijms24076137/s1.
