# Supplementary figures and images for: Genome-Wide Characterization and Expression Analysis of Transcription Factor Families in Desert Moss Syntrichia caninervis under Abiotic Stresses
Source: Int J Mol Sci. 2023 Mar 24;24(7):6137. doi: 10.3390/ijms24076137 (PMC10094499; doi:10.3390/ijms24076137)

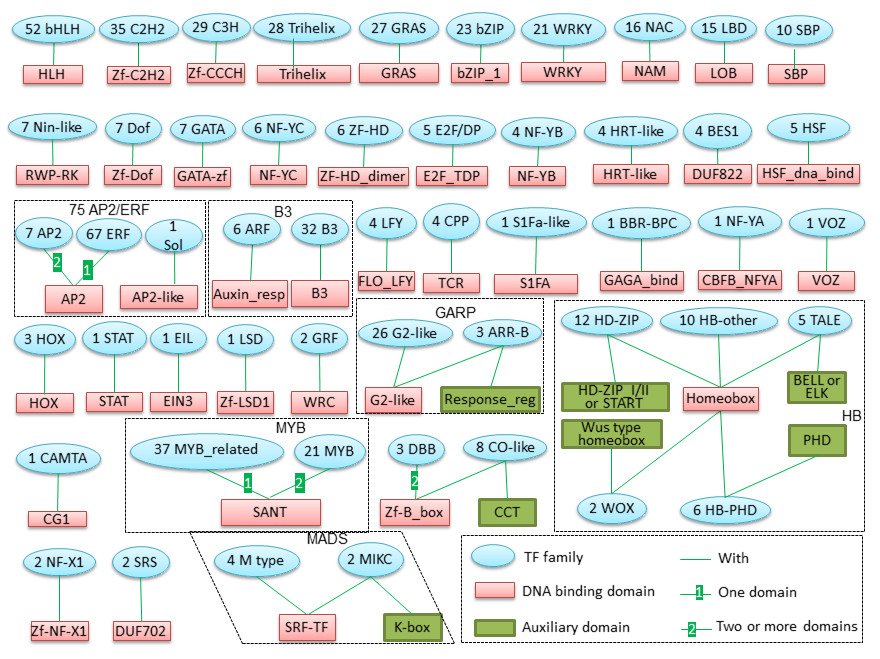

Supplement: Supplementary file 1 [file ijms-24-06137-s001.zip › Figure S1.tif]
